# ARMIA: A Sensorized Arm Wearable for Motor Rehabilitation

**DOI:** 10.3390/bios12070469

**Published:** 2022-06-29

**Authors:** Gabriel J. Garcia, Angel Alepuz, Guillermo Balastegui, Lluis Bernat, Jonathan Mortes, Sheila Sanchez, Esther Vera, Carlos A. Jara, Vicente Morell, Jorge Pomares, Jose L. Ramon, Andres Ubeda

**Affiliations:** Human Robotics Group, University of Alicante, 03690 San Vicente del Raspeig, Spain; gjgg@ua.es (G.J.G.); aaj24@alu.ua.es (A.A.); gbg12@alu.ua.es (G.B.); lbi5@alu.ua.es (L.B.); jme29@alu.ua.es (J.M.); ssr54@alu.ua.es (S.S.); evm45@alu.ua.es (E.V.); carlos.jara@ua.es (C.A.J.); vicente.morell@ua.es (V.M.); jpomares@ua.es (J.P.); jl.ramon@ua.es (J.L.R.)

**Keywords:** arm wearable, motor rehabilitation, biomechanics, electrophysiology

## Abstract

In this paper, we present ARMIA: a sensorized arm wearable that includes a combination of inertial and sEMG sensors to interact with serious games in telerehabilitation setups. This device reduces the cost of robotic assistance technologies to be affordable for end-users at home and at rehabilitation centers. Hardware and acquisition software specifications are described together with potential applications of ARMIA in real-life rehabilitation scenarios. A detailed comparison with similar medical technologies is provided, with a specific focus on wearable devices and virtual and augmented reality approaches. The potential advantages of the proposed device are also described showing that ARMIA could provide similar, if not better, the effectivity of physical therapy as well as giving the possibility of home-based rehabilitation.

## 1. Introduction

The number of people with motor disabilities has importantly increased in the last few years due to global aging and the general improvement of clinical care and health technology. For this reason, public and private health care systems are investing more in rehabilitation technologies. Recent events, such as the COVID-19 pandemic have stressed the importance of telerehabilitation both at home and at clinical facilities [1,2]. The use of telerehabilitation systems could avoid unnecessary physical contact between patients and therapists as well as enhance the rehabilitation process by increasing repeatability and intensity.

Upper limb motor impairment is one of the most limiting conditions for activities of daily living (ADLs), so an efficient rehabilitation is critical to recovering quality of life. This impairment can be caused by a variety of neuromuscular conditions such as stroke, spinal cord injury, neurodegenerative diseases, mistakes during surgery or aging. In the last few decades, a variety of robot-assisted technologies have been developed to outperform the efficacy of conventional manual therapies [3,4]. End-effector systems are the most typical devices to provide the patient with repetitive and continuous tasks. Examples of those are MIT-Manus [5], GENTLE [6] or, more recently, reachMAN2 [7]. Anthropomorphic devices such as upper limb exoskeletons are also common even though they are biomechanically more complex [8]. However, all these technologies are expensive and not affordable for particulars and for most rehabilitation centers.

Another key factor in upper limb motor rehabilitation is patient motivation. Studies on this behalf have been explored for many years to conclude that any motivational input increases the involvement of the patient in the requested motor tasks. For this purpose, a very common approach is the use of serious games, which are therapeutic leisure virtual activities to promote engagement. There are many applications of serious games to upper limb rehabilitation [9]. These applications can complement the use of assistive technologies. For instance, ArmAssist is a robotic device to improve arm mobility by using serious games [10], and VirtualRehab provides different interfaces for motor disabled individuals [11].

In addition to robot assistance, a good method to interact with these virtual environments is the sensorization of the patient. In the first stage, a common and simple way of performing this was using accelerometers to measure arm kinematics at the sagittal plane [12], but this did not allow inferring tridimensional information. With the development of modern inertial sensors (inertial measurement units, IMUs), it is now possible to characterize the whole arm kinematics with high precision. Moreover, kinematic metrics have a good correlation with conventional clinical scales, which provides an additional way of enhancing motor therapy [13]. Recent studies also show how ADLs can benefit from the application of this technology in rehabilitation [14,15]. Upper-limb motor rehabilitation can also benefit from the assessment of neuromuscular behavior. Surface electromyography (sEMG) allows for measuring electrical activity from the muscle contractions. This technique has been widely used to evaluate factors such as muscle fatigue [16] or movement coordination [17]. sEMG can also be used as input control for virtual environments by processing the activity of muscle contractions [18]. sEMG provides several advantages compared to other electrophysiological measurements, including non-invasiveness, real-time monitoring and on-site application with relatively affordable equipment.

In this paper, we present ARMIA: a sensorized arm wearable that includes a combination of inertial and sEMG sensors to interact with serious games in telerehabilitation setups. This device reduces the cost of robotic assistance technologies to be affordable for end-users at home and at rehabilitation centers. Hardware and acquisition software specifications are described together with potential applications of ARMIA in real-life rehabilitation scenarios. Similar wearable devices have already been applied in the telerehabilitation of specific body parts such as fingers [19] or the elbow [20]. In this case, ARMIA will provide rehabilitation to the whole arm by tracking upper-limb kinematics through inertial sensors. It also includes the real-time assessment of neuromuscular function by extracting meaningful biomarkers of motor control such as muscular fatigue. ARMIA is meant to be combined with virtual activities defined to track the whole rehabilitation process and optimize it based on the recorded physiological data.

## 2. Hardware

**Figure 1:**
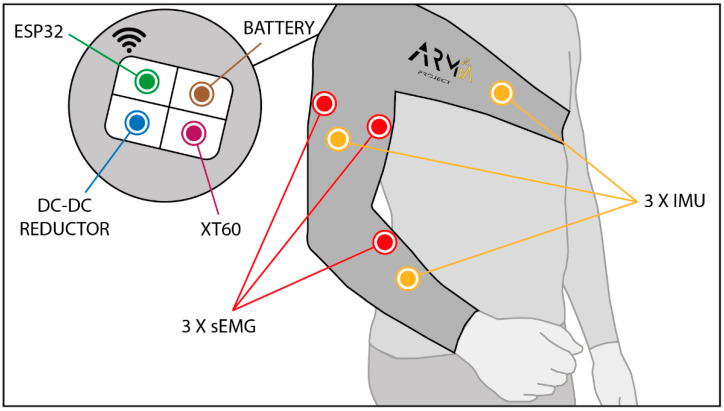
General appearance of the ARMIA wearable technology. A textile arm sleeve holds the inertial and sEMG sensors and is fastened to the chest for better fixation. The back of the wearable holds the battery and the microcontroller that communicates with the computer or device where serious games are running.

ARMIA is a sensorized sleeve that allows measuring kinematics and muscular activity during upper limb movements (Figure 1). In order to determine arm kinematics, three inertial sensors are placed on the kinematic chain: one on the thorax (reference), one on the arm and one on the forearm. These last two sensors provide arm link orientation and elbow and shoulder joint angles. ARMIA does not provide wrist orientation, but it is possible to obtain cartesian coordinates of the arm end effector (hand).

Muscular activity is measured from three of the main muscles in charge of upper limb movements: *biceps*, *triceps* and *pronator teres*. These sensors are used to determine the signal amplitude (contraction force) and other factors such as muscular fatigue. The instrumentation of these sensors is critical, as the bipolar electrodes must be placed parallel to the muscle fibers close to the middle of the muscle belly, and the reference electrode must be firmly in contact with the skin.

By considering these previous aspects, all hardware elements were selected according to a series of implementation requirements for the sleeve. The sleeve needs to be easily adapted to a variable morphology, and sensors, electronics and connections should be protected from external harm. All these components were selected from low-cost and lightweight options to make the device affordable and ergonomic. The different parts of ARMIA are described next.

### 2.1. Microcontroller

In order to acquire all analogical signals from the device, an ESP32 board (Espressif Systems) was chosen. ESP32 is an SoC (System on Chip) that can perform as a complete standalone system to connect with multiple peripheral devices, including a variety of sensors, and has very low energy consumption. This element communicates wirelessly with the computer in order to acquire the data from the inertial and EMG sensors.

### 2.2. Sensors

Muscular information (sEMG signals) is acquired via surface bipolar electrodes using MyoWare sensors (Sparkfun). These sensors provide a compact approach to the analysis of muscular activity and include the possibility of recording both raw signals and preprocessed signals (rectification and linear envelope). Kinematic information is acquired via Inertial Measurement Units (IMUs) (model LSM6DS33). Each sensor provides accelerometer and gyroscope data and allows measuring both orientation and relative position of arm joints in space.

### 2.3. Battery

The overall power consumption of each element was analyzed to determine the proper power supply to all the circuitry and sensors. The power supply needs to guarantee wireless communication for a period of several hours. A two-cell Li-Po battery was finally selected due to its small size and weight. This type of battery also provides a higher capacity compared to other options and allows the system to work for 3 h at full charge. Additionally, a DC–DC reductor was included to adapt the battery voltage output (7.4 V to 5 V) to feed the microcontroller and all the connected components.

### 2.4. Structural Elements

**Figure 2:**
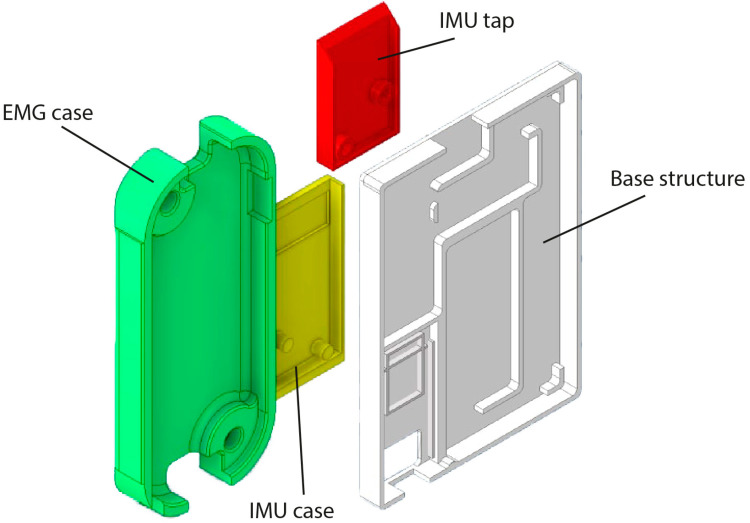
CAD models of the structural elements, including base structure and sensor holders.

Three different elements were designed to support the electrical components of the sleeve (Figure 2). CAD models were designed using Autodesk Inventor and 3D printed in PLA. The first element is the base structure for the main circuit, which includes the cover. The two other elements are supporting carcasses for the EMG and inertial sensors. The base structure can be closed with no need for screws and houses the ESP32 microcontroller, the battery and the DC–DC reductor. Every element is visible and easy to access. For the EMG sensors, the carcass improves the contact of electrodes with the skin and has a free space on the bottom to easily place the sensor over the skin. Finally, the IMUs carcass was designed to completely encapsulate the electronics and only give access to connection pins.

### 2.5. Garment

In order to design an adequate garment to hold the sensors, morphological restrictions were applied in the selection of the textile and size of the garment. The garment consists of two main elements: the sleeve that covers de arm up to the shoulder and the chest band to fix the wearable and hold the main electronics (see Figure 1). On the shoulder, there is a connecting textile. The primary material is neoprene with additional textile elements.

For the initial prototype, we assumed an average sleeve length of 32 to 33 inches (around 81–84 cm) for men and between 30 and 31 inches (around 76–79 cm) for women. The first prototype was built under this assumption. However, a wider range of the garment size will be developed to cover different populations, including children.

## 3. Software

ARMIA software is structured into three different levels. The low-level layer establishes the physical communication protocols between the different sensors and the microcontroller and the wireless communication between the microcontroller and the computer. The medium-level layer manages the data acquisition and storage from the sensors. Finally, the high-level layer develops processing and visualization modules.

### 3.1. Communication Layer

This layer manages communication between the sensors and the ESP32 microcontroller and between ESP32 and the computer. IMUs use an I2C communication protocol. The original library was modified to allow working with multiple addresses without the need for a multiplexor. EMG sensors already offer an analog output, so they are directly connected to the microcontroller’s analog inputs. Communication with the computer is performed wirelessly using a WiFi UDP protocol due to its simplicity. This protocol allows a high-frequency information exchange necessary for real-time analysis of kinematic and muscular data.

### 3.2. Acquisition Layer

Once all sensor information is captured by the ESP32 and sent to the computer, a database manages all the data through a visual interface. This interface offers three options: “Capture”, “Query” and “Exit”. “Capture” starts data acquisition through the UDP socket and stores all the data in the database. It also implements user management options, including parameters such as user name or capture time. Additionally, the received data are sent to another port to allow an external application to, for instance, visualize them. “Query” is used to search for previously stored information. It is possible to search data by user name or date. Once selected, CSV files are generated with all the requested data. Finally, “Exit” ends the execution of the acquisition interface.

### 3.3. Processing and Visualization Layer

**Figure 3:**
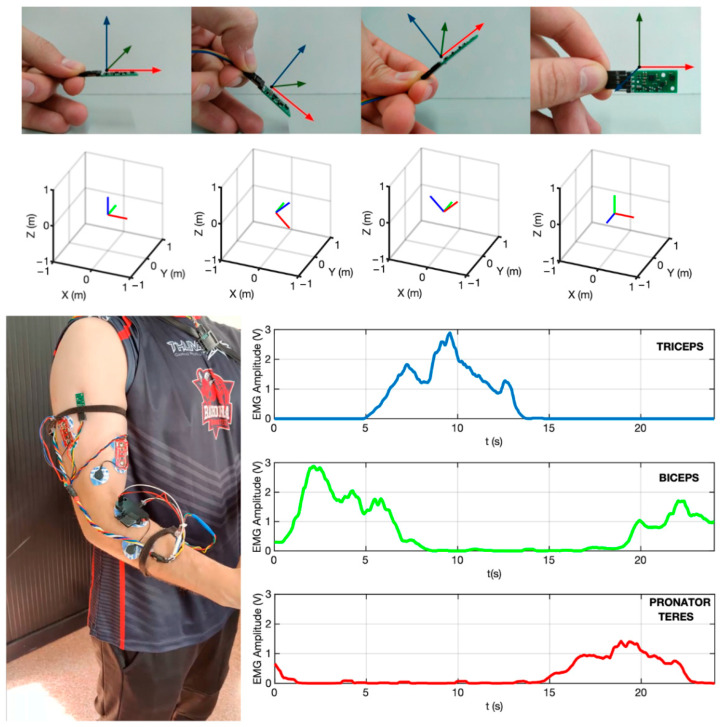
Current ARMIA prototype showing actual location of sensors (**bottom-left**). EMG signals obtained from the muscles of interest (**bottom-right**). Inertial sensor measurements (**top**).

Measurements from the different sensors are sent to the computer and then processed and visualized in real time. In the case of inertial sensors, the processing deals with information from both the gyroscope and the accelerometer included in the IMU. Accelerometers are not sensitive to external accelerations not originating from gravity, while gyroscopes have a significant drift in time. In order to reduce these errors, a complementary filter was designed following Equation (1):

(1) $$\theta_{angle}=\alpha *\left(\theta_{angle}+\omega_{gyro}*dt\right)+\left(1-\alpha \right)*a_{acc}$$

where $\theta_{angle}$ is the estimated orientation angle, α is the filter’s coefficient, $\omega_{gyro}$ is the angular velocity from the gyroscope, dt is the differential of time (period) and $a_{acc}$ is the angle obtained from the accelerometer measurements. The complementary filters have a short-term dependence on the gyroscope, which is precise but uses accelerometer data to avoid drift in the long term. However, the IMUs need to be calibrated before each use to avoid undesirable measurements. Figure 3 (top) shows an example of an IMU in different orientations. For each orientation, a real 3D visualization was computed. On the other hand, EMG signals only need to be scaled for visualization (Figure 3, right). As mentioned in the previous section, Myoware sensors provide both raw and processed data. Figure 3 also shows the prototype of the ARMIA device, including the three recorded muscles (biceps, triceps and pronator ceres) and the three IMUs (thorax, arm and forearm).

## 4. System Validation

In order to validate the first prototype of ARMIA, several experiments were performed. The main goal was to evaluate how accurate are the recorded signals from the prototype sensors and how they can be translated into meaningful inputs for the virtual activities. A description of some of the current virtual activities proposed to test ARMIA is presented in Section 5.3. These activities have simple inputs, such as flexo-extension angle or muscular amplitude, which are the two main parameters evaluated in the experiments.

### 4.1. Experiments

Five subjects participated in the study (all male; age 24.6 ± 6.4 years old; height 177 ± 7 cm). None of them had neuromuscular disorders that could affect the recordings, and all of them signed the corresponding informed consent according to the declaration of Helsinki. Data protection documents were also signed. Two experiments were performed:

- Experiment 1 consisted of tracking a specific angle profile by performing arm flexion movements. The duration of the run was 60 s. Four different angle levels were evaluated: 0° (full extension), 30°, 60° and 90° (arm flexed). Transitions between levels were interpolated to maintain the continuity of the movement;
- Experiment 2 consisted of performing five short contractions of the *biceps* muscle at full capacity during a recording of 40 s. Participants were given timing feedback and asked to perform the contractions after the first 5 s. For evaluation purposes, the first and last 5 s were removed.

### 4.2. Results

**Figure 4:**
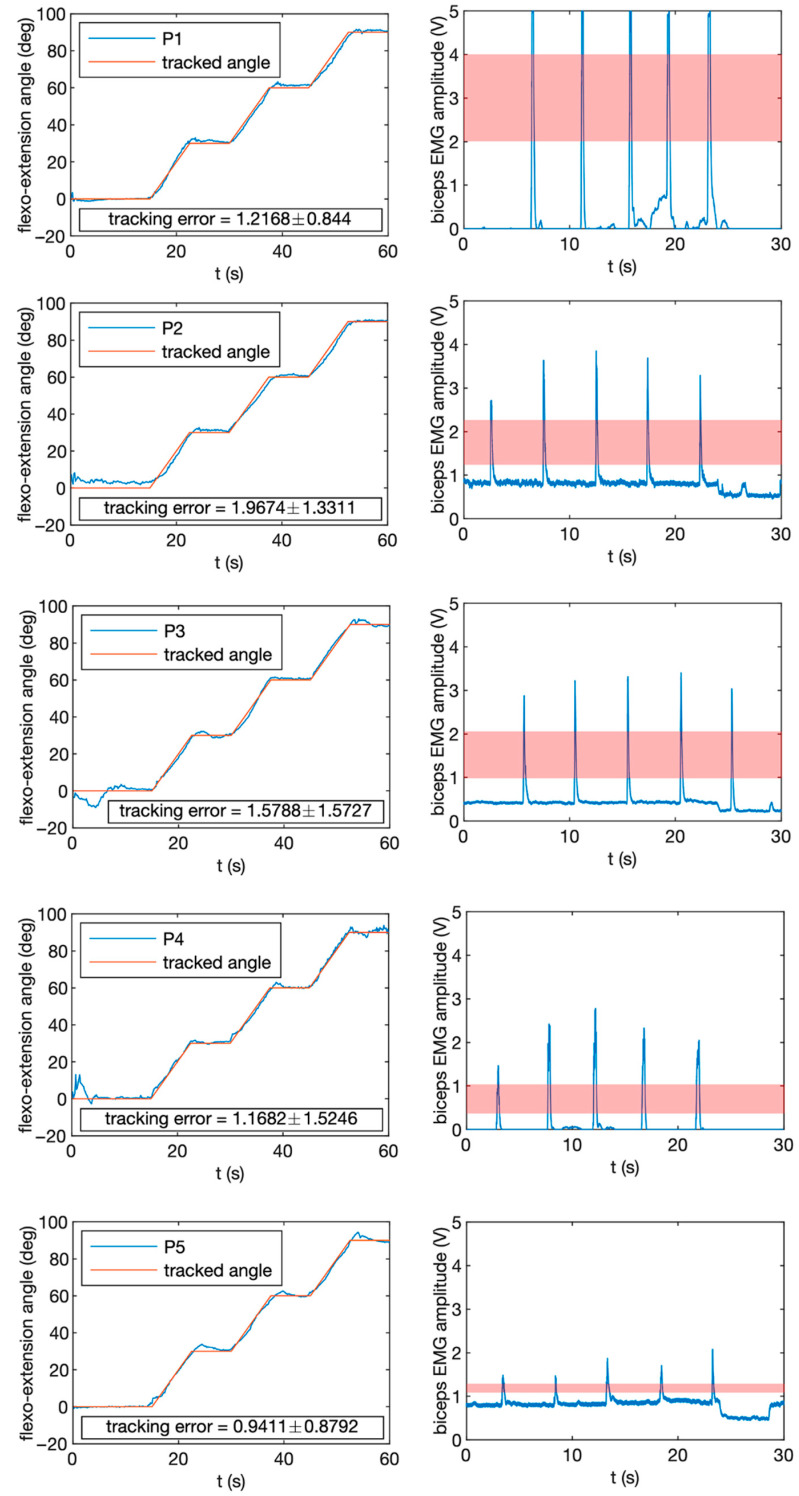
Results obtained for participants P1–P5. Experiment 1: Tracked angle vs. recorded angle, including mean tracking error. Experiment 2: Performance of 5 biceps contractions and proposed threshold range.

Figure 4 shows the results of the system validation. The inertial sensors are very reliable, and participants were capable of tracking with a very small error the angle profile provided visually (Figure 4, left). The tracking error was always below 2 deg and, in most of the cases, close to 1 deg or even below. Participants had some difficulty slowing down and adapting to constant angles, but the readjustment was very fast. EMG recordings are, in general, less reliable due to the intrinsic problematics of biosignal measurements. EMG signal is noisy, and electrodes need to be perfectly adjusted on the participant’s skin. However, our system provides enough quality to detect single contractions of measured muscles, in this case, the *biceps*. As observed in Figure 4 (right), most of the participants had a very clean muscle activation in each contraction. Only P2 and P5 had a higher base error. This was probably due to movements of the electrodes on the skin, bad fixation or the need for additional cleaning to increase electrode–skin conductivity.

## 5. Discussion

In this section, we addressed current issues in similar rehabilitation technologies and described the target population of our approach. We further analyzed other tools to enhance physical therapy that can be combined with our device. Particularly, we focused on the use of Virtual Reality (VR) and Augmented Reality (AR) to complement the rehabilitation activities with ARMIA. Finally, we described the in-progress and future work proposed to improve the whole rehabilitation system.

### 5.1. Comparison with Current and Previous Technology

Previous technologies are commonly relying on robotic support to enhance rehabilitation intensity and dose. Rehabilitation robots have optimal repeatability, multiple programs, high reliability and are ideal for large hospitals, but their cost is unbearable for small clinical centers or patient associations, not to mention their use by particulars. These devices are also not portable, and rehabilitation setups are fixed to a particular setting with specialized personnel, not allowing their use for telerehabilitation. In order to solve these limitations, we proposed the use of wearable technology such as ARMIA, which has proven to be a less cumbersome and economical way to approach physical rehabilitation [21,22].

Many wearable alternatives have been developed in recent years to provide ways of rehabilitating upper limb function from home. An interesting approach is GameRehab, a telerehabilitation system that combines serious games and wearable kinematic sensors [23]. Other systems also explore the use of accelerometers to detect arm movements [24,25]. An alternative for kinematic recognition is the use of strain sensors as in [26]. In another study, muscular information from sEMG sensors was used to detect upper-limb fatigue levels [27,28]. The use of haptic feedback has also been a matter of interest in some studies to show quantitative improvements in children with neuromotor disorders [29]. What seems to be clear is that there is a consensus that this kind of device offers improvements in physical rehabilitation [21,22,30,31].

The proposed device ARMIA combines the advantages of previously cited works as it includes both kinematic information and muscular information. The initial aim of this device is to use EMG and kinematic inputs in a simultaneous but independent way. However, future developments will explore the possibility of sensorial fusion, for instance, by inferring correlations between EMG activity and kinematics. This will allow obtaining a better understanding of how motor control behaves during a rehabilitation task and allow readapting the physical therapy in a more efficient way and improve functional outcomes. ARMIA also offers compatibility with VR and AR applications, as well as virtual on-screen applications. The advantage of this combination with external activities (serious games) was further analyzed in Section 5.3. One of the benefits of the device is ergonomics, as all the sensors are intended to be inserted in a textile to fit comfortably on the user’s arm.

### 5.2. Clinical Scope

ARMIA is suitable for a wide range of people with motor limitations, although it could be complemented with cognitive tasks if combined with other tools (see Section 5.3). The main groups that benefited from this technology are:

- People who have suffered recent brain damage due to stroke, traumatism or any other condition;
- People with neurodegenerative or neuromuscular diseases of any kind who need regular physical therapy;
- Elderly people with mobility problems in the upper limb and in need of maintaining physical activity;
- People affected by post-traumatic or post-surgical complications in arms or hands that require rehabilitation.

### 5.3. Current and Future Developments

From the sensor data, ARMIA software extracts kinematic and muscular parameters to be used with serious games compatible with Virtual Reality (VR) and Augmented Reality (AR) technology. VR is a simulated experience that incorporates different sources of sensory feedback, including auditory, visual or, in some cases, haptic stimuli. In the case of AR, this simulation directly overlays the real world, providing computer-generated perceptual information that complements our view of the surrounding environment. Examples of applications of these technologies to a wide range of motor pathologies can be found in a variety of studies which include approaches with virtual environments [32,33,34,35,36,37,38,39], immersive environments using head-mounted devices [40,41,42,43,44] or the more recent use of AR [45,46,47,48,49,50].

**Figure 5:**
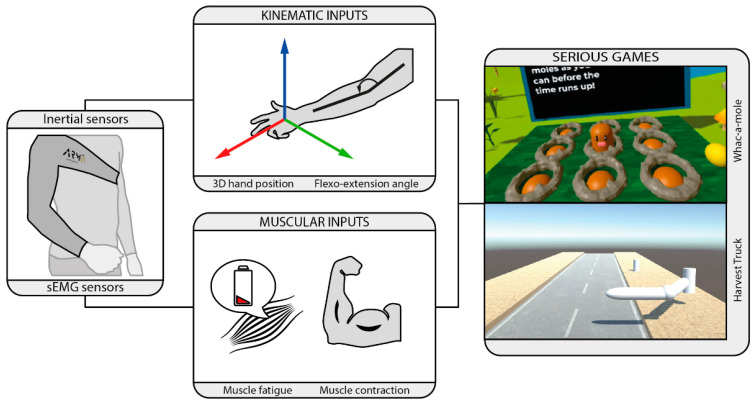
Control inputs for the gamification activities. Kinematic data (3D hand position and flexo-extension angle) and muscular data (fatigue and contraction) are used to command serious games and evaluate motor performance.

Figure 5 describes how the extracted kinematic and muscular parameters could be translated into a meaningful control input for the games. To manage movement in the virtual environment, users can provide control in different levels of complexity, from single joint angle to bidimensional or tridimensional Cartesian position of the hand. The first proposed input is arm flexion and extension angle by comparing arm and forearm orientation. Three-dimensional Cartesian coordinates of the hand are obtained with respect to the reference sensor (thorax) by resolving the direct kinematics of the arm. The bidimensional position is achieved by extracting projections of the hand 3D coordinates on the transverse plane. This is a relatively short set of kinematic inputs but very suitable for common motor rehabilitation activities in daily therapy. Muscular inputs can provide onset for special actions or determine force level. In-game actions could be related to a sustained force, for instance, to achieve a particular goal, or related to short muscle activations, to control additional actions while performing the correct arm movements. Moreover, parameters such as muscle fatigue could be considered to decrease difficulty at some points.

By considering these possible control inputs, two different games are being developed for the ARMIA device. These games are particularly focused on planar movements and are a good starting point for the device validation:

- *Whac-a-Mole:* this game focuses on the rehabilitation of reaching tasks on the transverse plane. The players grab a virtual ball to smash the moles that appear on the screen by reaching their position and hitting them. The score increases on each correct hit. Players have different possibilities for hitting depending on the level of impairment and pathology. One option is to use an external input such as a button to hit the moles or use arm co-contraction to hit the moles at the precise moment. If players have big movement limitations on the hand, the hit can be performed by using the so-called dwell click, which sets the ball to take action automatically when the ball stops moving for a certain amount of time in a certain range to the target. The game will have different difficulty levels that dynamically change depending on players’ performance, or that could be customized by therapists. This difficulty can be based on increasing the number of moles that show up per minute, decreasing the time they are visible, increasing the score required to advance to the next level or including cognitive challenges such as hitting a mole with a particular appearance to obtain extra points.
- *Harvest Truck:* this game only takes two control inputs from the wearable: the flexion and extension angle and an action input corresponding to the contraction force level measured on one of the recorded muscles. The players are on the back of a harvest truck traveling on a rural road. On the side of the road, different vegetable boxes of different weights are waiting for pickup. Players must extend the arm to grab the boxes and apply a different amount of contraction force depending on the weight or size of the boxes to be capable of moving them. In this case, in-game difficulty levels change by adding or removing total boxes, increasing box weight or changing the distance to the harvest truck. The score will be computed depending on the number and weight of the collected boxes on each level.

Future developments for the application of ARMIA will be focused on the evaluation of how motor rehabilitation can be benefited from the use of the proposed gamified activities and how our device can provide the necessary control inputs to allow both clinical rehabilitation and home-based telerehabilitation in an entertaining but effective manner.

## Data Availability

Data is available upon request. Please contact: andres.ubeda@ua.es.
